# Voltammetric Ion-Selective Electrodes in Thin-Layer Samples: Absolute Detection of Ions Using Ultrathin Membranes

**DOI:** 10.1021/acs.analchem.3c04224

**Published:** 2024-01-05

**Authors:** Yujie Liu, Gastón A. Crespo, María Cuartero

**Affiliations:** † Department of Chemistry, School of Engineering Science in Chemistry, Biochemistry and Health, KTH Royal Institute of Technology, SE-100 44 Stockholm, Sweden; ‡ UCAM-SENS, Universidad Católica San Antonio de Murcia, UCAM HiTech, Avda. Andres Hernandez Ros 1, 30107 Murcia, Spain

## Abstract

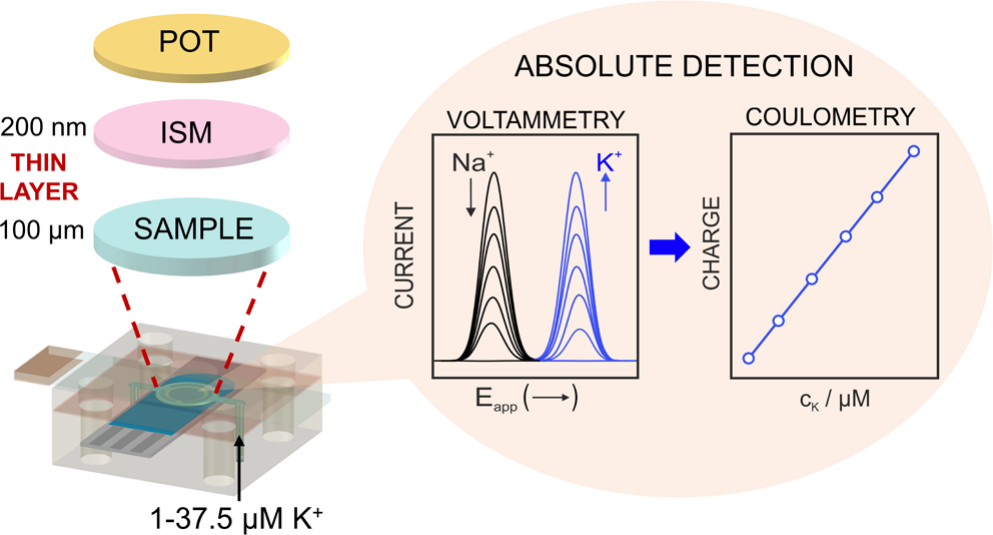

Calibration-free sensors are generally understood as analytical tools with no need for calibration apart from the initial one (i.e., after its fabrication). However, an “ideal” and therefore “more restricted” definition of the concept considers that no calibration is necessary at all, with the sensor being capable of directly providing the analyte concentration in the sample. In the electroanalysis field, investigations have been directed to charge-based readouts (i.e., coulometry) that allow for concentration calculation via the Faraday Law: The sample volume must be precisely defined and the absoluteness of the electrochemical process in which the analyte is involved must be ensured (i.e., the analyte in the sample is ∼100% converted/transported). Herein, we report on the realization of calibration-free coulometric ISEs based on ultrathin ion-selective membranes, which is demonstrated for the detection of potassium ions (K^+^). In essence, the K^+^ transfer at the membrane–sample interface is modulated by the oxidation state of the conducting polymer underlying the membrane. The accumulation/release of K^+^ to/from the membrane is an absolute process owing to the confinement of the sample to a thin-layer domain (thickness of <100 μm). The capacity of the membrane expressed in charge is fixed to ca. 18 μC, and this dictates the detection of micromolar levels of K^+^ present in ca. 5 μL sample volume. The system is interrogated with cyclic voltammetry to obtain peaks related to the K^+^ transfer that can be treated charge-wise. The conceptual and technical innovative steps developed here made the calibration-free detection of K^+^ possible in artificial and real samples with acceptable accuracy (<10% difference compared with the results obtained from a current-based calibration and ion chromatography). The charge-based analysis does not depend on temperature and appeared to be repetitive, reproducible, and reversible in the concentration range from 1 to 37.5 μM, with an average coulometry efficiency of 96%.

Modern society is in demand of rapid and reliable chemical sensing platforms capable of providing decentralized measurements of target analytes. In this context, ion-selective electrodes (ISEs) have gained increasing research interest due to their unique simplicity and versatility when applied to this societal request.^1^ However, the necessity of frequent calibration to maintain accuracy expectations when implemented in the corresponding gadgets (e.g., wearable sensors and submersible probes) may restrict their use in real-world scenarios.^2,3^ Coulometry in thin-layer samples emerged as a promising strategy to realize calibration-free electrochemical sensors.^4^ With the sample confined to a thin-layer space of less than 100 μm thickness, the complete conversion/transport of the analyte is achievable.^5^ Through the application of a proper perturbation, it is possible to obtain an electrochemical response related to such a conversion or transport and calculate the associated charge. Ultimately, the charge is converted to concentration through the Faraday law, which requires knowing the sample volume with high precision.^6^ Considering ∼100% efficiency, such a process to obtain the concentration does not require any calibration.

Early investigations in the field of coulometry in thin-layer samples for ion detection dated from 2002 and were focused on ion-transfer voltammetry at the interface between two immiscible liquids.^7,8^ Osakai and co-workers used a membrane filter to create a nitrobenzene–water interface over a thin channel built on a Ag/AgCl plate: complete electrolysis was accomplished for the interfacial transfer of the tetramethylammonium ion.^7^ Sánchez-Pedreño et al. presented a method for chronocoulometric measurements using a membrane-based ISE in a microfluidic cell and following a four-electrode configuration with ohmic drop compensation.^8^ A double potential step was synchronized to the passage of the sample plug through the ISE surface, allowing for the detection of the tetraethylammonium ion (TEA^+^) at the micromolar levels. Later on, Kihara and co-workers reported on the selective coulometric detection of hydrophilic ions (K^+^, Ca^2+^, and Mg^2+^) with a porous Teflon tubing containing the thin-layer sample (sandwiched between the wall of the tubing and an internal Ag/AgCl wire) and immersed in turn into an organic phase comprising the corresponding ionophore.^9^ Upon the application of a constant potential, the ion analyte was transported from the sample layer to the organic phase. Despite all of these pioneer studies shedding light on the absolute detection of ions, their applicability was restricted to fundamental purposes.

Advantageously, polymeric (e.g., polypropylene) tubings can be doped with appropriate ion receptors to implement ISEs as coulometric systems. This was demonstrated for the exhaustive detection of K^+^, Ca^2+^, protamine, NO_3_^–^, and NO_2_^–^.^10−16^ A flat configuration was also realized for the detection of TEA^+^ and K^+^, with a sample drop (volume of 1 μL) being placed between a planar Ag/AgCl reference electrode and the ISE, made of plasticized Teflon and doped poly(3,4-ethylenedioxythiophene) (PEDOT) as the ion-to-electron transducer.^17^ In any case, the application of a constant potential generates the ion transfer at the membrane–sample interface, resulting in a current response that can be integrated for charge calculation. Notably, undesired nonfaradaic processes may contribute to the current signal, requiring the application of a second potential pulse for background correction.^11^

The sample interrogation with cyclic voltammetry (CV) revealed interesting directions. The calibration-free coulometric detection of halides (Br^–^, Cl^–^, and I^–^) in diverse samples was realized.^18,19^ Their electrodeposition in silver wires (tubular cells) and plates (planar cells) in the form of silver salts manifested in three charge-dependent peaks. Furthermore, the application of the CV–coulometry tandem was demonstrated for the determination of K^+^ at millimolar levels and the tetrabutylammonium ion (TBA^+^) at micromolar levels using an inner-filling solution ISE equipped with a K^+^-selective membrane that was ca. 50 μm-thick.^20^ Despite being thinner than in traditional potentiometric ISEs, such a membrane thickness is likely responsible for a coulometric efficiency of <85% at the selected interrogation time (very low scan rates ranging from 2 to 10 mV s^–1^), e.g., 84 and 50% for 50 and 500 μM TBA^+^ concentrations, respectively. Mass transport (i.e., diffusion-dependent process) in the membrane is indeed a limited factor in the working mechanism underlying the coulometric response.

In the context of coulometric ISEs, there has been a clear trend of decreasing the thickness of the ion-selective membrane (ISM) to establish fast responses when facing electrical perturbations. Bobacka and co-workers reported on coulometric all-solid-state ISEs composed of doped PEDOT and H^+^-selective membranes with a thickness of 5–10 μM.^21^ Spin-coated membranes presented a faster amperometric response than that of drop-cast membranes: the thinner the membrane, the faster the response. A decrease in the membrane resistance was found to contribute to such a behavior. The charge readout of the current–time curves was proportional to pH from 6 to 9.5, with a calibration graph being necessary for its analytical application. Even thinner membranes (∼230 nm) were also explored but this time in connection to poly(3-octylthiophene), labeled herein as POT.^22^ The application of a potential sweep in the positive direction promotes the oxidation of POT, which generates a charge disbalance that ends up in the expelling of cations (i.e., ion-transfer process, IT) from the membrane to the solution for the maintenance of electroneutrality.^23^ Each IT manifests in a voltammetric peak that can be considered charge-wise for analytical purposes, e.g., to detect Ag^+^ and K^+^ both at nanomolar and micromolar levels depending on the applied protocol.^24,25^

To the best of our knowledge, coulometric all-solid-state ISEs based on thin ISMs have not yet been investigated when coupled to thin-layer samples. Effectively, the research questions are: How attainable is an exhaustive and thus calibration-free approach? Is the concept suitable to analyze real samples? One may intuit that when a thin-layer regime (i.e., mass transport is not a limiting factor because it is not a diffusion-dependent process) applied to both the sample and the ISM is reached, new analytical opportunities will arise. This is indeed herein investigated. Accordingly, we present the concept of calibration-free coulometric all-solid-state ISEs. To achieved that, (i) the ISE is based on a redox active element (such as POT) connected to an ultrathin ISM, (ii) the sample is confined to a thin-layer domain by means of a planar microfluidic cell, (iii) the IT at the sample–membrane interface ultimately occurring upon the polarization of the ISE is of exhaustive nature (i.e., ∼ 100% efficiency), and (iv) the ISM is designed with the capacity to host increasing charge of K^+^ (as the proof of concept) coming from the sample. This paper reports on the establishment of the operational protocol as well as the investigation of the voltammetric, coulometric, and calibration-free features of such a system, finally applying it to K^+^ detection in different samples. The presented calibration-free sensor may be tailored for the determination of any ion, being also applicable to the indirect analysis of (bio)molecules that can be derivatize to ions, just by correlating the exchange capacity of the membrane with the expected concentration in the sample. Moreover, the developed microfluidic format of the sensor is in principle compatible with any decentralized application.

## Experimental Section

### Reagents, Materials, and Equipment

Aqueous solutions were prepared by dissolving the appropriate salts in deionized water (>18.2 MΩ). Potassium chloride solution (0.001 M), 3-octylthiophene (97%, OT), lithium perchlorate (>98%, LiClO_4_), polyurethane (PU, Selectophore), bis(2-ethylhexyl)sebacate (DOS), sodium tetrakis[3,5-bis(trifluoromethyl)phenyl]borate (NaTFPB), potassium ionophore I (Valinomycin), sodium chloride (99.999%, NaCl), potassium chloride (99.5%, KCl), tetrahydrofuran (>99.9%, THF), and acetonitrile (anhydrous, >99.8%, ACN) were purchased from Sigma-Aldrich. Absolute ethanol (99.5%) was acquired in VWR. Indium tin oxide (ITO)-coated glass slides (10 mm × 35 mm × 1.1 mm, surface resistivity <10 Ω/sq, transmittance >83%) were sourced from Zhuhai Kaivo Optoelectronic Technology. Screen-printed platinum electrodes (Pt-SPE, DRP-550) were purchased from Metrohm Dropsens. The PTFE tape and the copper tape were purchased from RS Components. The silicon rubber was supplied by the Junying CNClathing company.

CV measurements were performed by using a VIONIC potentiostat controlled with INTELLO software (supplied by Metrohm). An 850 Professional ion chromatography (IC) instrument equipped with a Metrosep C 6–150/4.0 column, conductivity detector, and 863 Compact Autosampler (injection volume of 10 μL) was employed to validate the real sample analysis (sample eluent of 2.5 mM HNO_3_, flow rate of 0.9 mL/min). Calculations were accomplished with MATLAB_R2018b software.

The lake water and seawater samples were obtained from the Lappkärret lake and Lilla Värtan strait in Stockholm, Sweden. The samples were collected and filtered first with regular filter paper (Whatman, diameter of 150 mm) and then with 0.2 μm-pore size filters coupled to syringes to remove large particles. The standard 0.001 M KCl solution was purchased from Sigma-Aldrich. All the samples were stored in a fridge prior analysis. For electrochemical measurements, all of the samples were diluted in 10 mM NaCl solution at different volumetric ratios. The NaCl background ensured the appropriate conductivity of the very diluted samples. For IC, the samples were directly analyzed after filtration without any dilution.

### Preparation of the ITO-POT-Membrane Electrode

The ITO electrodes were cut to the size of 10 mm × 35 mm and then cleaned by ultrasonification with ethanol, followed by a rinse with water. Notably, substrates other than ITO could have been used, such as glassy carbon or gold electrodes. A PTFE tape was applied to the ITO surface to define the active surface area as a circular region with a diameter of 8.00 ± 0.01 mm. Electrical contact was established by using copper tape. The synthesis of poly(3-octylthiophene) (POT) was carried out via electropolymerization in a solution, the composition of which was 0.1 M 3-octylthiophene and 0.1 M LiClO_4_ in ACN. After degassing with nitrogen for 15 min, the POT film was generated using CV within a potential range from 0 to 1.5 V, scan rate of 100 mV s^–1^ and applying two scans. This procedure generates a POT film of a charge of ca. 370 μC. The film was then discharged at 0 V for 120 s. A platinum counter electrode and a homemade Ag/AgCl (wire) reference electrode were employed during the experiment. The synthesized POT film was subsequently immersed in ACN (30 min), then in THF (10 s) and finally dried with a smooth flow of nitrogen. POT films synthesized following this procedure were previously characterized by surface imaging, XPS, spectroelectrochemistry, ellipsometry (ca. 50 nm thickness), and other techniques.^22,25,26^ The cocktail to obtain the ISM contained 20 mg of PU, 20 mg of DOS, 0.8 mg of NaTFPB, and 2 mg of potassium ionophore I dissolved in 2 mL of THF. The POT-ITO-membrane electrode was prepared by spin-coating 25 μL of this solution (1500 rpm, 60 s) using a 6808P spin coater (PI-KEM). This procedure provides a membrane thickness of ca. 230 nm on top of the ITO-POT electrode.^22^ The TFPB^–^ charge in the membrane is calculated to be ca. 18 μC, and thus, the POT is in excess with respect to the TFPB^–^ element.

### Preparation of the Thin-Layer Microfluidic Cell

**Figure 1:**
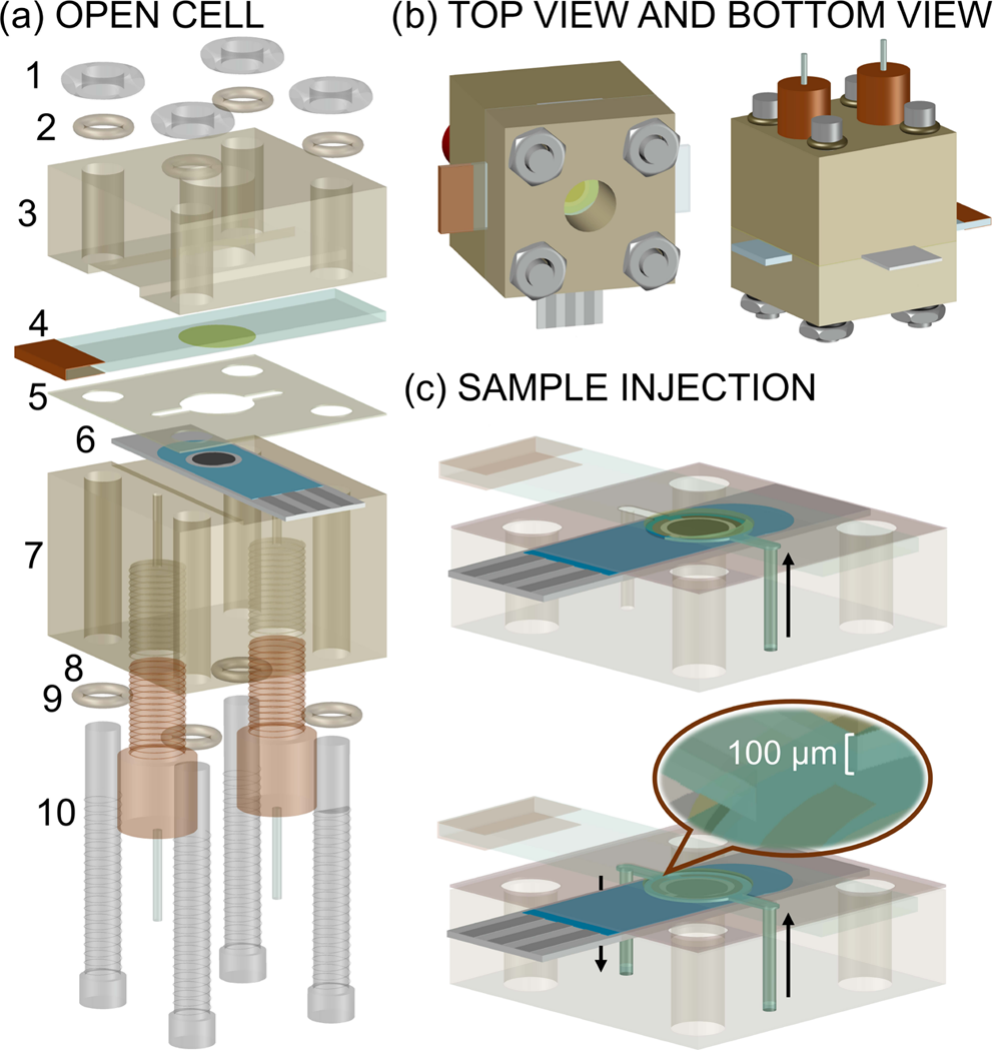
(a) Thin-layer microfluidic cell composed of (1) hex nuts, (2 and 9) O-ring, (3) ITO electrode holder, (4) ITO-POT-membrane electrode (with a copper tape as a connector), (5) silicon rubber spacer (thickness of 100 ± 5 μm), (6) Pt-SPE, (7) SPE holder, (8) inlet and outlet, and (9) screws. (b) Top and bottom views of the assembled cell. (c) Injection of the thin-layer sample into the microchannel.

The individual components of the microfluidic cell are shown in Figure 1a. The ITO-POT-membrane acted as the working electrode and was positioned in a PTFE holder containing in turn a hole of 4 mm diameter in the center for the visualization of the thin-layer sample by naked eyes (to confirm the absence of any bubble). There is a second PTFE holder designed for Pt-SPE to fit on it. The Ag and Pt elements of this SPE were utilized as the reference and counter electrodes, respectively. The thin-layer compartment for the sample was defined by a rubber-based microfluidic channel (thickness of 100 ± 5 μm) sandwiched between the ITO-POT-membrane electrode and Pt-SPE. The microfluidic channel is in turn connected to the inlet and outlet of the cell. Once the screws and nuts are tightened, the cell is securely sealed and ready for sample injection with a peristaltic pump. The top view and bottom view of the assembled microfluidic cell are depicted in Figure 1b. An illustration of the sample injection through the rubber-based microchannel is shown in Figure 1c. A real photograph of the experimental setup is displayed in Figure S1.

## Results and Discussion

### Mechanism and Its Premises

This work investigates the absolute nature of IT events across ultrathin ISMs linked to thin-layer samples. For that purpose, we designed the experimental setup shown in Figure 1, which provides a microfluidic system to introduce and replace the sample to be analyzed with the ITO-POT-membrane electrode. In essence, the application of a potential sweep in the positive direction generates POT^+^, which ultimately promotes a cation transfer (the IT) at the membrane–sample interface, driven by electroneutrality conditions. The IT manifests in a voltammetric peak that can be considered charge-wise for analytical purposes. When this process occurs in connection to a thin-layer sample, it can be of absolute nature, meaning that the cation is totally transferred across the membrane–sample interface.

**Figure 2:**
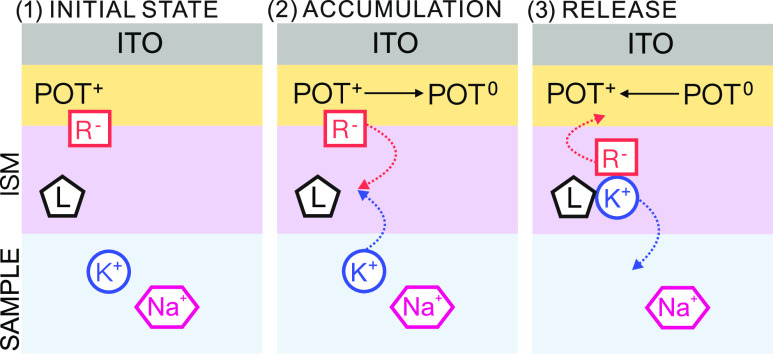
Illustration of the working mechanism in the thin-layer cell, considering the anodic part of the CV experiment. Step 1: Application of 1 V was carried out along sample injection. Step 2: Fast equilibration of the ITO-POT-membrane with the thin-layer sample. Step 3: Anodic sweep potential for the detection of cations.

To avoid the presence of any cation in the membrane that may interfere with the absolute nature of the IT event, before any electrochemical measurement, the ITO-POT-membrane electrode was initially polarized at 1 V while the sample is being injected into the cell (with a peristaltic pump). Once the sample is injected, the potential and pump are stopped and a cyclic voltammetry (CV) protocol that generates the IT at the membrane–sample interface is applied. The detection of ions will occur in the anodic part of the CV. More in detail, the initial applied potential of 1 V produces the POT film in its oxidized form (POT^+^), doped with the TFPB^–^ present in the membrane (from the cation exchanger, Na^+^TFPB^–^). Driven by electroneutrality maintenance, this caused the countercation Na^+^ (and indeed any cation present in the membrane) to be expelled to the solution, so as the membrane is totally “clean” of cations. Accordingly, all the cations are expelled from the membrane while the sample is being injected, as illustrated in Step 1 in Figure 2.

Then, because of the thin-layer configuration of both the sample and the membrane (i.e., no mass transport limitation because it is not a diffusion-dependent process), it is assumed that a fast equilibrium will be established between the ITO-POT-membrane system and the sample just at the beginning of the potential sweep. It is hence expected that neutral POT^0^ is present in the system, with the concomitant cations’ accumulation in the membrane from the sample solution (Step 2 in Figure 2). Remarkably, it has been demonstrated that very thin ISMs equilibrate very fast (in a matter of milliseconds) once in contact with a sample solution, resulting in the exchange of ions at the sample–membrane interface.^24^ The cations entering the membrane should pair the TFPB^–^ released from the POT film lattice to the membrane upon its reduction. Due to the presence of the ionophore, K^+^ is the preferred cation by the membrane in such a way that the K^+^ in the sample is exhaustively depleted via accumulation into the membrane. When available (depending on the K^+^ concentration in the sample), the rest of cationic positions in the membrane will be covered by Na^+^ (or other nonpreferred cations in the background/matrix) in the sample.

Upon the application of the anodic part of the CV experiment, from a certain potential, the conversion from POT^0^ to POT^+^ being doped with TFPB^–^ is generated. This results in the expulsion of the cations accumulated in the membrane back to the solution (Step 3 in Figure 2), which gives rise to a voltammetry peak used herein for the detection of K^+^. Later, the cathodic part of the CV will generate in turn the reverse process, so that successive scans result in the described accumulation/release of cations from/to the membrane and to/from the solution, i.e., the IT events.

Regarding the achievement of absoluteness in the IT, the (positive) charge capacity of the membrane and sample volume must be appropriately selected to provide such a condition. In our system, the charge capacity of the membrane (i.e., the maximum positive charge from the sample solution that the membrane can lodge) was fixed to ca. 18 μC. This was achieved by the membrane composition described in the Experimental section, i.e., using a membrane cocktail containing 0.85 mg of NaTFPB in the plasticized polymeric matrix. As established elsewhere, the cationic positions that can be occupied in the membrane are fixed by the cation exchanger amount.^23^ On the other hand, considering a sample volume of 5.0 ± 0.3 μL (provided by the channel in the microfluidic cell designed here), the 18 μC in the membrane would translate into a sample K^+^ concentration of ca. 37.5 ± 2.4 μM (applying the Faraday’s law). In other words, the ITO-POT-membrane would be able to embed the K^+^ charge present in a 37.5 μM-concentrated sample solution, considering 100% depletion. Accordingly, an analytical methodology based on this concept will be applicable until such a concentration. Higher concentrations could be reached by a compromising change of the charge capacity of the membrane and the sample volume, while keeping the thin-layer behavior of both.

### Investigation of the Voltammetric Behavior of the ITO-POT-Membrane Electrode in the Thin-Layer Cell

**Figure 3:**
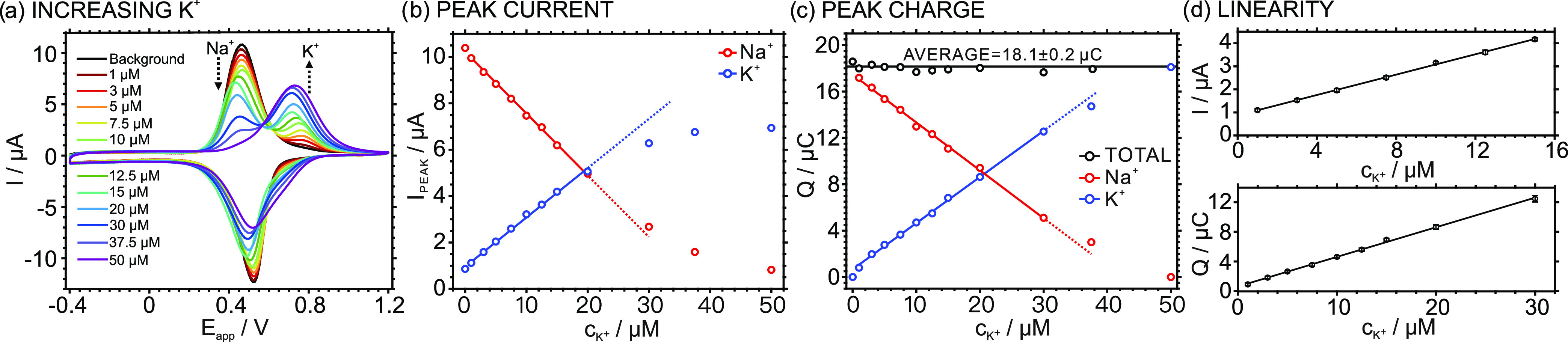
(a) CVs at increasing KCl concentrations with a 10 mM NaCl background. Scan rate: 100 mVs^–1^. (b) Plot of the peak currents for Na^+^ and K^+^ transfers versus the K^+^ concentration in the sample. (c) Plot of the peak charges for Na^+^ and K^+^ transfers versus the K^+^ concentration in the sample. (d) Linearity of the peak current and charge with the K^+^ concentration in the sample (*n* = 3).

First, the voltammetric behavior of the ITO-POT-membrane electrode was explored for increasing K^+^ concentrations in the range of 1–50 μM in 10 mM NaCl background solution. The experimental protocol was as follows: flowing the sample for 150 s at 100 μL/min while applying a potential of 1 V, stopping the flow and the applied potential, and recording the CVs (three scans, from −0.4 to 1.2 V). This was repeated for each concentration. Figure 3a presents the corresponding CV results for the third scan. Notably, the K^+^ entrance from the solution to the membrane was not entirely achieved in the first scan at the highest concentrations, as concluded from the slightly lower K^+^ peak (at ca. 720 mV) compared to the second and third scans. However, this effect was not realized for the lowest concentrations (see Figure S2 in the Supporting Information for a comparison of subsequent scans observed for 3 and 30 μM).

Attending to the anodic part of the CV, the voltammogram obtained in the NaCl background electrolyte (without any added K^+^) presented only one peak at 420.7 mV, attributed to the transfer of Na^+^ (the only cation in the solution). After K^+^ was introduced in the thin-layer sample, another peak appeared at 718.3 mV. This is therefore ascribed to the transfer of K^+^. The appearance of the voltammetric peak for the Na^+^ transfer at a lower peak potential than that for K^+^ was indeed expected: a higher energy is required to release K^+^ than for Na^+^ from the membrane, since K^+^ retention in the membrane is thermodynamically favored owing to the presence of the selective ionophore.^23^ Then, the increasing concentration of K^+^ in the sample solution translated into an increase in the voltammetric peak of K^+^, coupled with a decrease in the Na^+^ peak. In essence, there is a competition between Na^+^ and K^+^ present in the sample to occupy the positive vacancies available in the membrane. Despite the ionophore preferring K^+^ over Na^+^, this preference is only manifested from a certain K^+^ concentration, especially considering the higher amount of Na^+^ in the background of the experiments with respect to K^+^.^25^ Thus, increasing K^+^ concentrations in the solution manifest in an increasing K^+^ peak, while the Na^+^ peak decreases, as the total charge is always the same but divided between these two cations and the K^+^ preference to enter the membrane over Na^+^ enhances at increasing K^+^ concentrations. When the K^+^ concentration in the sample was 50 μM, only the peak for K^+^ was present, since all the positive vacancies in the membrane are entirely occupied by K^+^.

The relationship between the peak current and charge of the anodic waves for Na^+^ and K^+^ transfers with increasing K^+^ concentration in the sample solution was further analyzed. To calculate the charge associated with two overlapped peaks, a method considering the baseline correction of the voltammograms followed by peak deconvolution via Gaussian fitting was first performed (see an example in Figure S3 in the Supporting Information**)**. Notably, the method considers the experimental scan rate to convert the applied potential into time and an integration interval of 0.1 s. Then, the charge for each peak was calculated by integrating the corresponding fitting curve. Figure 3b,c presents the trends of the peak current and charge with increasing K^+^ concentration in the sample, respectively. The current for the Na^+^ peak decreased, while that for the K^+^ peak increased. In essence, more K^+^ accumulates in the membrane when more K^+^ is present in the sample. Due to the preference of the membrane for K^+^ over Na^+^ because of the presence of the ionophore, K^+^ accumulation over Na^+^ always occurs.

The total charge remained constant at an average value of 18.1 ± 0.2 μC (rather coinciding with the TFPB^–^ charge set in the membrane: 18 μC) while being distributed between the two IT peaks. Accordingly, the charge lost in the Na^+^ transfer corresponded well to the charge gain in the K^+^ transfer at increasing K^+^ concentration in the sample solution. Moreover, this trend displayed excellent linearity in the concentration range from 1 to 30 μM, slightly losing the linearity from the 37.5 μM-concentrated solution. Remarkably, this concentration is close to the maximum concentration that has been estimated that the membrane is able to lodge, considering the K^+^ transfer to be exhaustive (∼100%). Then, for the higher K^+^ concentration that was tested (50 μM), the charge calculated for the K^+^ peak totally coincided with the total charge available in the membrane (i.e., initially presented by the Na^+^ peak), meaning that all the positive sites were previously occupied by K^+^ and, thus, the peak magnitude cannot further increase. Importantly, the K^+^ peak is not expected to change in terms of current and width (and consequently in the charge) from the saturation situation (i.e., from the 37.5 μM concentration). However, in our experiments, we found a slight change in the charge going from 37.5 to 50 μM. Effectively, the real saturation concentration may be between these two concentrations, showing a small deviation from the theoretical value.

The charge–concentration linearity appeared over a wider concentration range than the current–concentration one, which is in principle logical acknowledging that the response mechanism is based on a series of interconnected charge transfer processes, as explained above. In any case, both linearities were confirmed in triplicate experiments with three equal ITO-POT-membrane electrodes. An excellent between-electrode reproducibility was observed in both the voltammetric response (Figure S4 in the Supporting Information) and the linear fittings of the peak currents and charges (Figure 3d, error bars). The curve calculated for the peak current within the K^+^ concentration range from 1 to 15 μM was *I*_K_ = 0.221*c*_K_ + 0.873; *R*^2^ = 0.999, with *I*_K_ expressed in μA and *c*_K_ in μM. The curve calculated for the peak charge within the K^+^ concentration range from 1 to 30 μM was *Q*_K_ = 0.402*c*_K_ + 0.604; *R*^2^ = 0.998, with *Q*_K_ expressed in μC and *c*_K_ in μM. The intercept of this linear regression was close but not equal to the ideal zero, which is presumably due to the presence of traces of K^+^ in the background electrolyte or the microfluidic cell.

All the discussion carried out until this point has been based on the anodic part of the CV. Regarding the cathodic part, the peak for the K^+^ transfer was not as well-defined as that in the anodic part, resulting in a high degree of overlapping with the peak for the Na^+^ transfer (see Figure 3a). Nonetheless, the total integrated charges from the anodic and cathodic waves corresponding to the same voltammetric scan were significantly close between them (Table S1 in the Supporting Information). For example, in the CV of the background electrolyte, the charges of the Na^+^ peak in the anodic and cathodic parts were calculated to be 18.58 and 18.70 μC, respectively. Then, for all of the tested K^+^ concentrations, an average difference of 1% was calculated. This value pointed out the adequate reversibility of the electrochemically related events and ultimately the IT processes occurring at the sample–membrane interface in the system under study.

### Investigation of the Absoluteness Nature of the Ion Transfer Process and the Possibility for a Coulometric Readout

**Figure 4:**
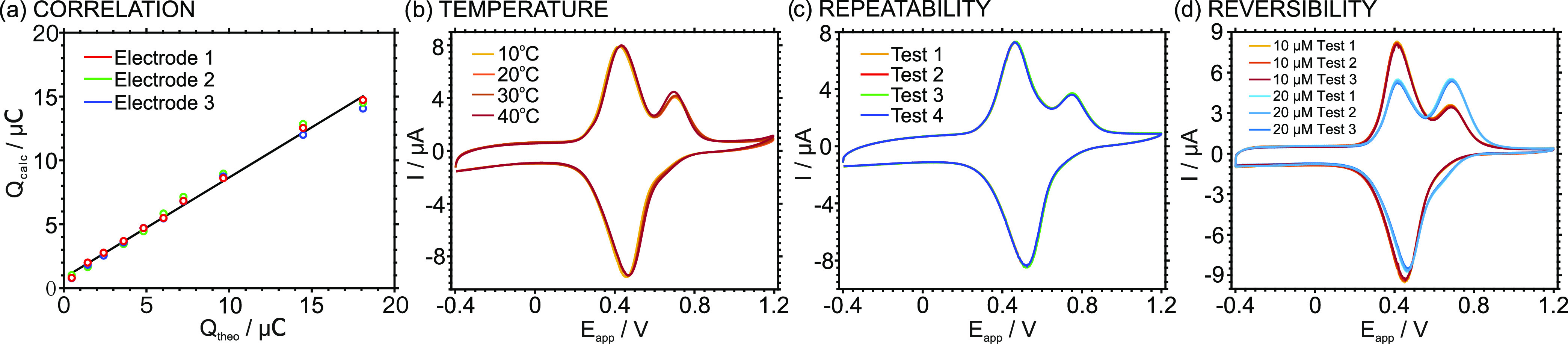
(a) Correlation between the calculated charges (*n* = 3, *Q*_calc_) and the theoretical charges (*Q*_theo_) present in thin layer samples containing increasing K^+^ concentration from 1 to 37.5 μM. (b) CVs at increasing temperatures (10–40 °C) for 10 μM KCl in a 10 mM NaCl solution. (c) CVs of four repeated tests using the same ITO-POT-membrane electrode in flour plugs of 10 μM KCl in 10 mM NaCl solution. (d) Reversibility of the electrode response following the sequence of 10 → 20 → 10 → 20 → 10 → 20 μM K^+^ concentration in 10 mM NaCl solution. Scan rate: 100 mVs^–1^.

The thin-layer behavior of the system was confirmed: a linear correlation was found between the peak current of the anodic part of the CV and the scan rate, which ranged from 30 to 100 mV s^–1^ (Figure S5). Consequently, mass transport is not a limiting factor for the current response, as the overall process is not a solution diffusion one. Then, it was investigated if the charge associated with the K^+^ transfer correlates with the K^+^ concentration in the sample solution in an exhaustive way and following the Faraday Law. Figure 4a shows the correlation between the theoretical charge and the experimental one obtained from the voltammetric peak at each concentration (*n* = 3 electrodes).

The theoretical charge was calculated considering the K^+^ concentration in the sample, the physical dimensions of the sample confinement, 100% K^+^ transfer from the solution to the membrane (in the fast accumulation step), 100% of the K^+^ transfer from the membrane to the solution (in the anodic potential sweep), and the Faraday Law. Accordingly, eq 1 was used
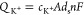
1where *Q*_K^*+*^_ is the charge associated with the K^+^ content in the thin-layer sample, *c*_K^*+*^_ is the concentration of K^+^ in the thin-layer domain (from 1 to 37.5 μM), *d*_s_ is the thickness of the sample (100 ± 5 μm), *A* is the area in where the sample is confined (50.2 ± 0.3 mm^2^), *n* is the moles of electrons transferred per mole of analyte (*n* = 1), and *F* is Faraday’s constant (96,485 C mol^–1^). Figure S6 presents the errors associated with the theoretical charges, calculated based on the uncertainties of the K^+^ concentrations in the sample and the volume.

Despite slightly higher deviations being found at the lowest K^+^ concentrations (i.e., 1 and 3 μM), the theoretical and experimental charges were found to rather coincide (Figure 4a). Indeed, a Pearson coefficient of 0.997 was calculated, indicating that a correlation between both charges exists (considering a threshold of 0.950 to discern the existence or absence of correlation). These results highlighted that charge-based measurements may be exhaustive and consequently reflect the K^+^ concentration in the sample. Moreover, the charge readout was temperature-independent and totally repeatable and reversible, as demonstrated in the following.

The effect of temperature variations on the charge response was evaluated by recording the CVs of the 10 μM KCl sample solution at 10, 20, 30, and 40 °C. The results are depicted in Figure 4b, with the four voltammograms being rather similar. Moreover, the charge under the K^+^ peak at each temperature presented a variation of just 0.5%. The independency of the charge with the temperature confirmed once more the thin-layer behavior of the system. To assess the repeatability, four subsequent CVs were conducted using the same electrode. Before each test, the sample (10 μM KCl in 10 mM NaCl solution) inside the thin-layer cell was replaced by pumping a new solution plug, the experimental protocol being fully repeated. The obtained voltammograms are presented in Figure 4c. There was a significant overlapping between the voltammograms, with the variation for the charge associated with the K^+^ transfer being less than 0.1%. Regarding reversibility, two samples containing 10 and 20 μM K^+^ concentrations were analyzed in consecutive cycles, increasing and decreasing the K^+^ concentration. Figure 4d shows the voltammograms observed in the sequence of 10, 20, 10, 20, 10, and 20 μM. Minor variations were identified: the charge of the K^+^ peak remained nearly constant for both concentrations (4.72 ± 0.09 μC for 10 μM and 8.81 ± 0.21 μC for 20 μM).

Considering all these results, the potential to develop a coulometry-based analytical approach was evidenced. Moreover, it could lead to a calibration-free methodology never reached before for voltammetric ISEs based on ultrathin membranes. Effectively, the charge under the K^+^ peak can be directly utilized to calculate the K^+^ concentration in the sample, under the established conditions. Accordingly, we evaluated the percentage of error when the charges read from the voltammograms are used to directly calculate the K^+^ concentration in the sample solution, based on eq 1. This percentage was, in turn, related to the coulometric efficiency or the percentage of absoluteness.

Table 1 collects the results obtained for the three identical electrodes. Concentration values rather similar as the theoretical ones were obtained. Remarkably, a coulometric efficiency of 96 ± 7% was estimated in the K^+^ concentration range from 5 to 30 μM, involving errors in the calculation of the K^+^ concentration of less than 15%. Larger differences were found in the lowest (1 and 3 μM) and highest (37.5 μM) concentrations. On the one hand, the determination of lower concentrations will be significantly influenced by any trace K^+^ that could remain in the microfluidic cell between measurements, leading to coulometric efficiencies higher than the 100%. Also, the preparation of the corresponding artificial samples is subjected from intrinsic errors, inducing an inherited deviation in the theoretical concentration (and charge). On the other hand, 37.5 μM concentration is close to the membrane charge capacity. Thus, the detection of higher K^+^ concentrations will benefit from the use of a membrane with a slightly higher NaTFPB concentration to increase the cation accumulation capacity.

**Table 1:** Coulometric Detection of K^+^ Concentrations In Thin-Layer Samples Containing Increasing K^+^ Concentrations in a NaCl Background Solution^a^

electrode 1		electrode 2		electrode 3						
*Q*_*K*^*+*^_ (μC)	*c*_K^+^_^calc^ (μM)	*Q*_K^+^_ (μC)	c_K^+^_^*calc*^ (μM)	*Q*_K^+^_ (μC)	*c*_K^+^_^calc^ (μM)	*Q*_K^+^_ (μC)^b^	*c*_K^+^_^calc^ (μM)^b^	*c*_K^*+*^_^added^ (μM)	efficiency (%)^c^	error in *c*_K^+^_^calc^ (%)^c^
0.81	1.7	0.78	1.6	1.02	2.1	0.87 ± 0.13	1.8 ± 0.3	1.0	180	80
1.97	4.1	1.83	3.8	1.65	3.4	1.82 ± 0.16	3.8 ± 0.3	3.0	125	25
2.77	5.7	2.55	5.3	2.60	5.4	2.64 ± 0.12	5.5 ± 0.2	5.0	109	9
3.68	7.6	3.58	7.4	3.35	6.9	3.54 ± 0.17	7.3 ± 0.4	7.5	98	2
4.70	9.8	4.71	9.8	4.45	9.2	4.62 ± 0.15	9.6 ± 0.3	10.0	96	4
5.49	11.4	5.46	11.3	5.84	12.1	5.60 ± 0.21	11.6 ± 0.4	12.5	93	7
6.83	14.2	6.80	14.1	7.14	14.8	6.92 ± 0.19	14.4 ± 0.4	15.0	96	4
8.61	17.9	8.73	18.1	8.96	18.6	8.77 ± 0.18	18.2 ± 0.4	20.0	91	9
12.54	26.0	12.00	24.9	12.85	26.6	12.46 ± 0.43	25.8 ± 0.4	30.0	86	14
14.72	30.5	14.06	29.2	14.51	30.1	14.43 ± 0.34	29.9 ± 0.7	37.5	80	20

a The K^+^ concentrations were calculated from the voltammetric charge via the Faraday Law. The coulometric efficiency was calculated from the known K^+^ concentration added to the sample. The error in the calculation of the K^+^ concentration was estimated considering that the methodology should present 100% coulometric efficiency.

b Average ± SD (*n* = 3 electrodes).

c Average (*n* = 3 electrodes).

Overall, these results advocate the coulometric use of the ITO-POT-membrane electrode to analyze water-based samples containing K^+^ at the micromolar level. Moreover, the K^+^ concentration in the sample can be obtained with acceptable accuracy (difference <15% in the K^+^ concentration range from 5 to 30 μM) without calibrating the electrode, just with the corresponding voltammogram.

Comparing the linear range of response herein observed for K^+^ (from 5 to 30 μM) with that reported by Yoshida et al. for K^+^ (100–800 μM) but also TEA^+^ (20–200 μM) in their laminated flat system (as described in the Introduction), our range is evidently narrower.^17^ As mentioned above, higher concentrations could have been reached by increasing the NaTFPB in the membrane. Moreover, compared to the laminated cell (it could be considered the closest system to the one developed here), the configuration, electrochemical protocol, and overall performance have been herein overcome in terms of (i) avoiding errors intrinsic to using tiny sample drops (i.e., 1 μL), (ii) canceling undesired interferences from the use of commercial inks, (iii) eliminating the influence of resistance in the voltammetric response, (iv) no need of subtracting the current profile corresponding to the blank to correct for the nonfaradaic process, and (v) unnoticed imprecisions in the system repeatability.

### Validated Analytical Application: Comparing Charge-Based (Calibration-Free) versus Current-Based (Calibration-Required) Measurements

Four water samples were analyzed: a reference KCl solution (CRM), lake water, seawater, and tap water. Notably, these samples were selected to demonstrate the appropriate operation of the system in terms of variety and matrix complexity. To cover the linear range of response found for the developed electrode, all of the samples were diluted with 10 mM NaCl. The K^+^ concentrations in the diluted samples were determined using two methods: (i) comparison of the peak current with a calibration graph and (ii) direct calculation based on the charge integrated from the K^+^ voltammetric peak. In both cases, each sample was analyzed with three equal electrodes. In addition, the undiluted samples were characterized by IC.

**Figure 5:**
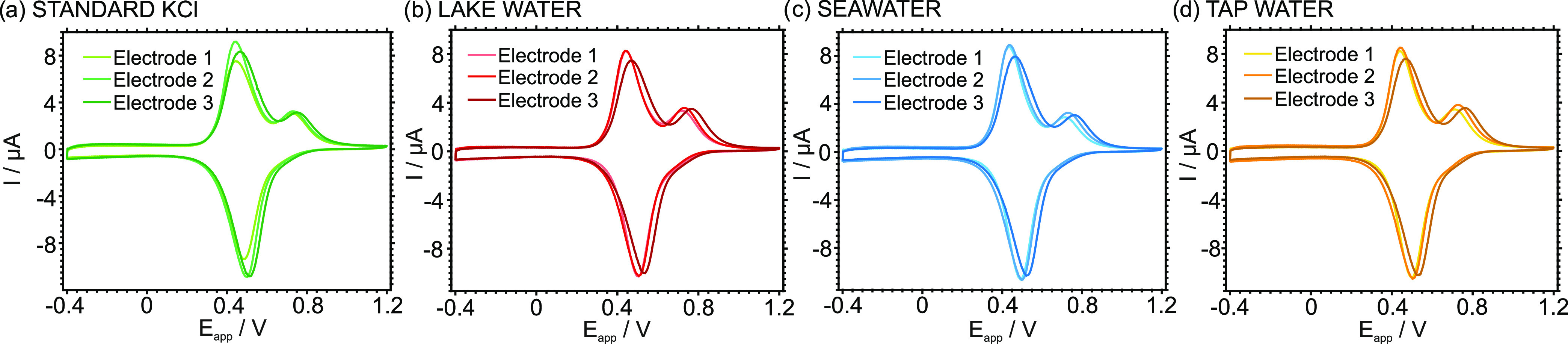
CVs observed for the diluted samples: (a) standard 0.001 M KCl solution, (b) lake water, (c) seawater, and (d) tap water. *n* = 3 equal electrodes. Scan rate: 100 mV^–1^.

Figure 5 shows the voltammograms for the four samples obtained with the three ITO-POT-membrane electrodes. All the samples displayed two peaks, where the first appeared at a lower potential (in the range from 425.5 to 477.6 mV) and was attributed to the cations different from K^+^ that are present in the sample and the second peak purely corresponded to K^+^ (appearing in the range from 718.3 to 757.8 mV). Indeed, the first peak will mainly correspond to the Na^+^ originally present in the sample and that added during the dilution. For other cations to contribute to such a peak, they must be present at relatively high concentrations (i.e., mM), as derived by the membrane selectivity profile.^23^

The formal peak potentials appearing in the voltammograms of the samples may slightly vary between electrodes, while the K^+^ peak current and charge displayed for the same sample were well maintained, as observed in Figure 5 and the standard deviations provided in Table 2. In contrast, slight variations in the current and charges corresponding to the background cation peak were found; this behavior is likely due to small differences present in the membrane composition contained in each of the tested electrodes. Effectively, variations in the NaTFPB content due to the preparation of the membrane cocktail (a weight-based process) and deposition on the ITO-POT electrode can induce such a fluctuation. It is worth mentioning that a difference in the Na^+^ peak is not supposed to impact the detection of K^+^. In essence, the K^+^ is prioritized to be accumulated in the membrane and will preferentially cover the positive vacancies (coming from the exchangeable part of the Na^+^TFPB^–^ initially present in the membrane), so that any change in the overall capacity of the membrane will affect the Na^+^ peak but not the K^+^ one.

**Table 2:** Detection of K^+^ Concentration in Water Samples and Comparison of the Results Obtained from Different Techniques

		ISE^a^				c	e	d	d
sample	dilution	peak current (I, μA)	c_K^*+*^_^calc,I^ (μM)	peak charge (Q, μC)	*c*_K^*+*^_^calc,Q^ (μM)	diff. I-Q (%)^b^,^c^	*c*_K^+^_^IC^ (μM)^e^	diff. I-IC (%)^b^,^d^	diff. Q-IC (%)^b^,^d^
standard KCl	1:100	3.16 ± 0.09	10.1 ± 0.3	5.49 ± 0.03	11.4 ± 0.1	11	–	–	–
standard KCl^g^	–	–	1015 ± 28^f^	–	1138 ± 6^f^	–	1044.78 ± 0.60	3	9
lake water	1:10	3.46 ± 0.12	11.6 ± 0.2	5.96 ± 0.03	12.4 ± 0.4	7	–	–	–
lake water	–	–	116 ± 2^f^	–	124 ± 4^f^	–	121.63 ± 1.60	5	2
seawater	1:100	3.08 ± 0.19	9.8 ± 0.2	5.22 ± 0.29	10.8 ± 0.6	10	–	–	–
seawater	–	–	979 ± 22^f^	–	1082 ± 59^f^	–	1090.70 ± 1.70	10	1
tap water	1:6	3.60 ± 0.20	12.2 ± 0.3	6.01 ± 0.12	12.5 ± 0.3	2	–	–	–
tap water	–	–	73 ± 2^f^	–	74.8 ± 1.5^f^	–	75.19 ± 1.32	3	1

a Average ± SD (*n* = 3 electrodes).

b Average (*n* = 3 electrodes).

c The percentage difference was calculated considering the average value of *c*_*K*^*+*^_^calc,I^ and *c*_K^*+*^_^calc,Q^ as the reference.

d The percentage difference was calculated considering the value of*c*_K^*+*^_^IC^ as the reference.

e Average ± SD (*n* = 3 measurements).

f The concentrations were calculated considering the dilution factors.

g Concentration reported by the manufacturer: 0.00095–0.00105 mol/L.

The average K^+^ concentrations calculated from the peak currents (*c*_K^*+*^_^calc,I^) and charges (*c*_K^+^_^calc,Q^) found for the diluted samples (Figure 5) are depicted in Table 2. Differences of less than 10% were found. Then, to be able to compare these results with those obtained in IC (*c*_K^*+*^_^IC^), the K^+^ concentration in the undiluted samples was estimated from the dilution factors considered in the preparation of the diluted samples. The results are presented in Table 2. Notably, the K^+^ concentration in the standard KCl solution was contained in the range reported by the manufacturer, whereas the K^+^ concentration in the sample from the Baltic Sea revealed a content within expectations (1–2 mM, since it presents a salinity slightly lower than that of common seawater).^27^ Overall, a good agreement was found with the IC technique, presenting differences of <10% for all the analyzed samples. Moreover, the K^+^ concentrations calculated with the charge through a calibration-free approach were slightly closer to the IC results than those calculated current-wise.

**Figure 6:**
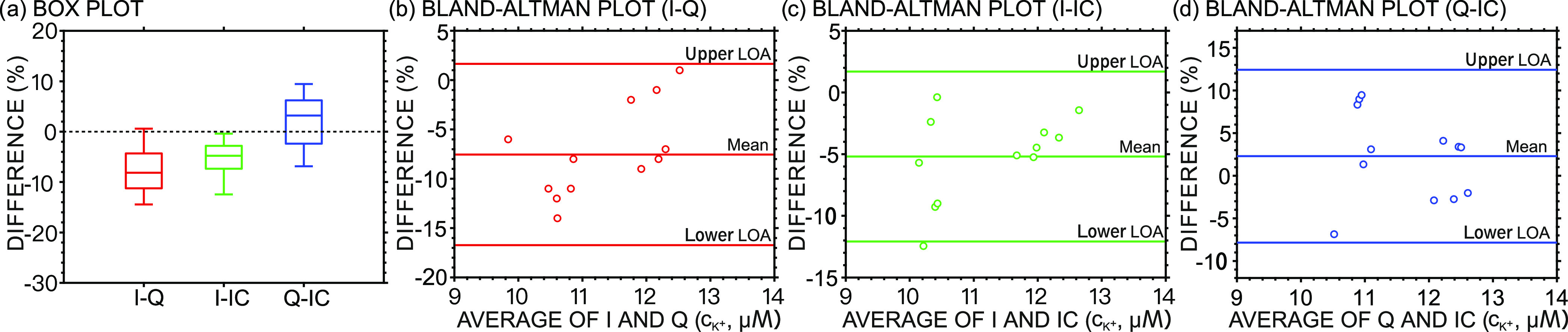
(a) Box plot for percentage differences in the detection of K^+^ obtained with current vs charge (I–Q), current vs IC (I–IC), and charge vs IC (Q–IC). The Bland–Altman plot of the percentage difference obtained with (b) current vs charge (I–Q), (c) current vs IC (I–IC), and (d) charge vs IC (Q–IC).

Figure 6a presents the box plot of the percentage differences between the three methods: current vs charge (I–Q), current vs IC (I–IC), and charge vs IC (Q–IC). As observed, the box portions for I–Q and I–IC are below the horizontal line at 0 (dashed), with the median values being −8.2 and −4.8%, respectively. This means that in most of the measurements, the values determined by a current-based readout were slightly lower than the charge-based readout and IC. Importantly, no statistically significant differences were found between the charge-based readout and IC. A Bland–Altman analysis was also conducted to assess the agreement among the three methods (Figure 6b–d). Percentage biases of −7.5 and −5.2% (from the plot of I–Q and I–IC) again indicated that the current-based readout may slightly underestimate the K^+^ concentration. The bias for Q–IC was calculated to be 2.3%, suggesting that the two methods are in good agreement. In all the cases, most of the data points fitted in the 95% limit of agreement, meaning that the differences between the three methods are relatively null.

Despite the results being acceptable and promising, it is important to realize any possible source of errors in the coulometric approach that may affect the overall accuracy. This includes (i) the protocol for charge integration (various methods could be evaluated and compared toward optimized results), (ii) the sample volume used in the Faraday law (this is defined by a rubber spacer that may undergo a deformation of 3% according to the manufacturer, and so the volume could slightly change and affect the charge–concentration conversion), and (iii) errors in the preparation of the diluted versions of the samples (the widening of the linear range of response would be desired in future work), among others. Accordingly, still, the developed calibration-free approach is prone to be improved toward an even better accuracy and coulometric efficiency.

## Conclusions

We have presented the implementation of a voltammetric ISE based on an ultrathin membrane selective for K^+^ with thin-layer samples. This has been achieved through the development of a microfluidic cell that allows for the precise confinement of the sample to a thickness of less than 100 μm and a volume of 5.0 μL. Such conditions permit the coulometric exploitation of the system in the K^+^ concentration range from 1 to 37.5 μM in the sample, matching the charge capacity of the membrane. Effectively, the cation exchanger concentration fixed in the membrane marks the positive charge vacancies that are available; then, because no mass transport limitation occurs in either the sample or the membrane, it is possible to exhaustively accumulate and later release (under a potential control) to/from the membrane the K^+^ present in the sample. The release process manifests in an anodic voltammetric peak, whose peak current and charge can be exploited for analytical purposes. Moreover, the use of the charge leads to a coulometric approach that does not need a previous calibration graph, i.e., the K^+^ concentration is estimated by a conversion from the charge by means of the Faraday law. The K^+^ charge is independent of temperature changes as well as excellently repetitive, reproducible, and reversible. Four real samples were successfully analyzed by the developed methodology. Remarkably, the results obtained from a coulometric basis and applying a calibration-free approach did not statistically differ from those found in IC, indicating an acceptable accuracy.

## Supporting Information Available

Calculated charges, consecutive CVs, baseline correction and peak deconvolution, reproducibility, and CVs at increasing scan rates (PDF)
