# Supplementary material for: Voltammetric Ion-Selective Electrodes in Thin-Layer Samples: Absolute Detection of Ions Using Ultrathin Membranes
Source: Anal Chem. 2024 Jan 5;96(3):1147–55. doi: 10.1021/acs.analchem.3c04224 (PMC10809245; doi:10.1021/acs.analchem.3c04224)
Supplement: Supplementary file 1 — ac3c04224_si_001.pdf [file ac3c04224_si_001.pdf]

Supporting Information for:

Voltammetric Ion-Selective Electrodes in Thin-Layer Samples. Absolute Detection of Ions Using Ultrathin Membranes

Yujie Liu[1], Gastón A. Crespo[1,2], María Cuartero[1,2,*]

[1]Department of Chemistry, School of Engineering Science in Chemistry, Biochemistry and Health, KTH Royal Institute of Technology, SE-100 44 Stockholm, Sweden
[2]UCAM-SENS, Universidad Católica San Antonio de Murcia, UCAM HiTech, Avda. Andres Hernandez Ros 1, 30107 Murcia, Spain

Table of Contents

Table S1	Integrated Charges of the Anodic and Cathodic Waves	S2
Figure S1	Photograph of the Microfluidic Setup	S3
Figure S2	Three Consecutive CVs Observed for 3 and 30 μM KCl Solutions	S3
Figure S3	Baseline Correction and Peak Deconvolution	S4
Figure S4	Calibrations with Three Electrodes	S5
Figure S5	IT Peaks at Different Scan Rates	S6
Figure S6	Correlation Between Q_{calc} and Q_{theo} (With the Associated Errors)	S7

Tables

Table S1. Comparison between the Integrated charges of the anodic and cathodic waves (Q_{anodic} and $Q_{cathodic}$) at increasing concentrations of KCl

c_{K^+} / µM	Q_{anodic} / µC	$Q_{cathodic}$ / µC	Difference / %
0	18.58	18.70	0.64
1	17.99	18.06	0.39
3	18.31	18.43	0.65
5	18.12	18.37	1.37
7.5	18.1	18.34	1.32
10	17.88	18.07	1.06
12.5	17.81	18.01	1.12
15	17.9	18.23	1.83
20	18.03	18.14	0.61
30	17.85	18.03	1.00
37.5	17.93	18.19	0.72
50	18.1	18.34	0.66

Figures

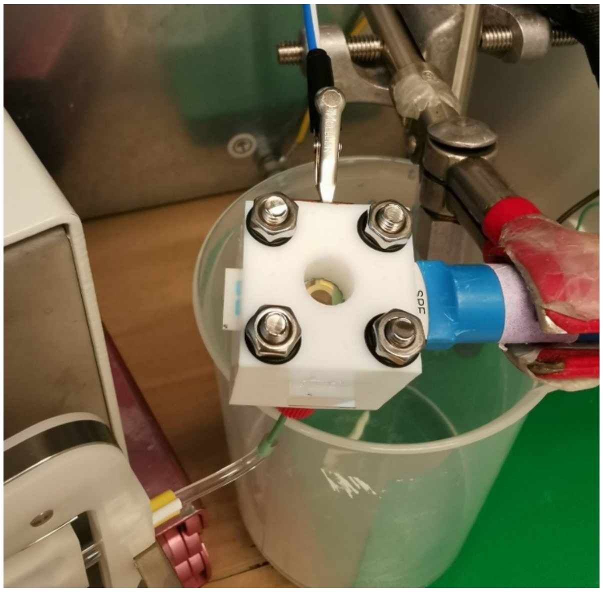

Figure S1. Photograph of the microfluidic setup based on the cell presented in Figure 1 in the main manuscript.

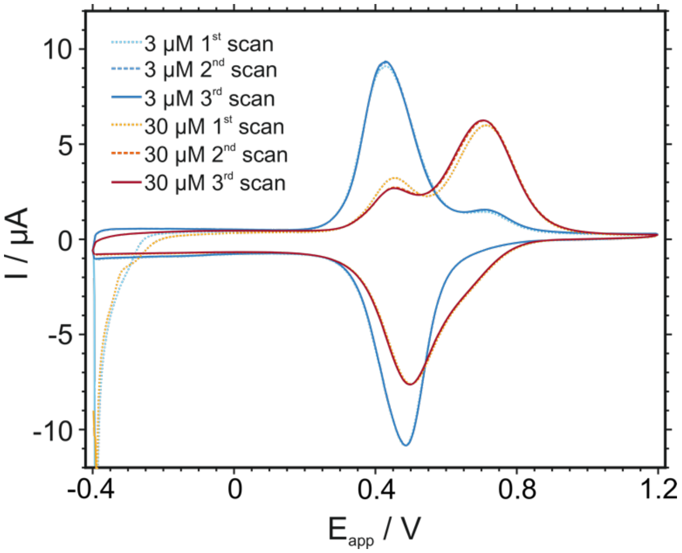

Figure S2. Three consecutive CVs observed for 3 and 30 μM KCl solutions with a 10 mM NaCl background. Scan rate: 100 mV s^{-1}.

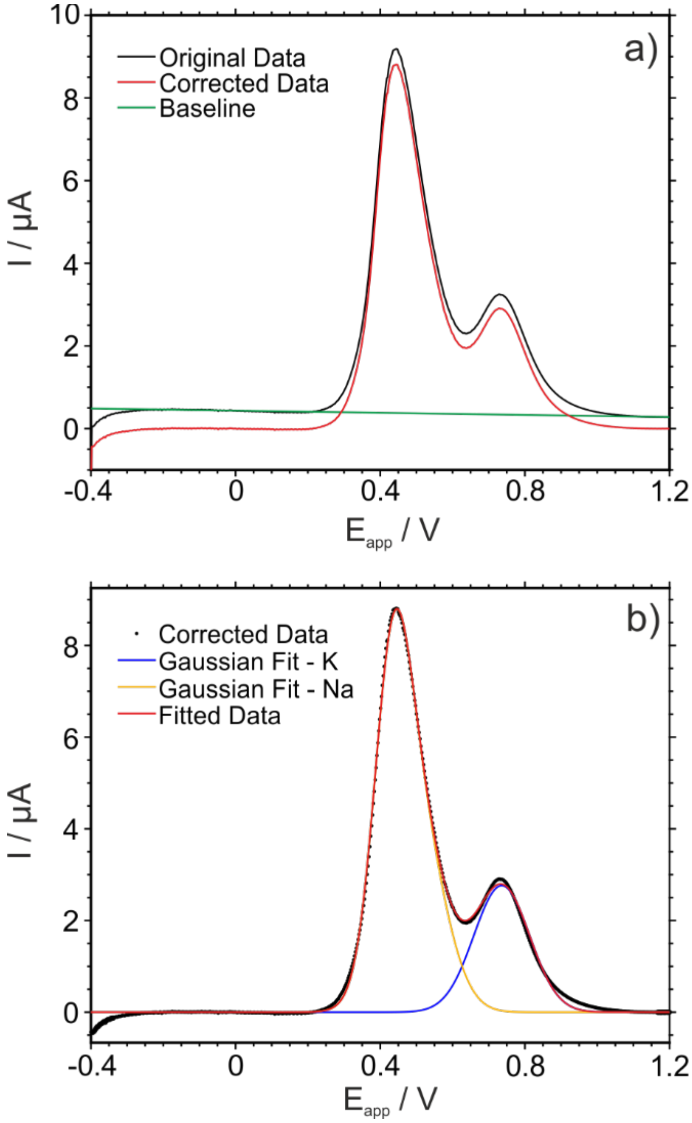

Figure S3. Illustration of (a) baseline correction and (b) peak deconvolution for the calculation of the charge for Na^+ and K^+ transfer peaks.

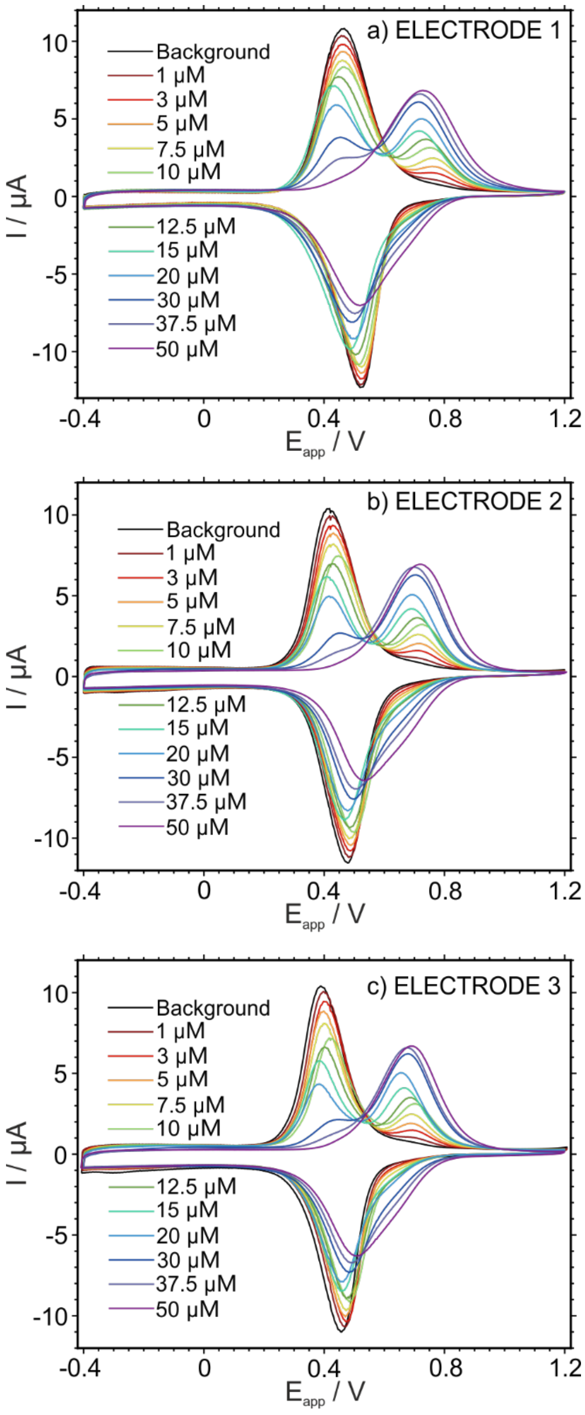

Figure S4. Cyclic voltammograms observed at increasing KCl concentrations in 10 mM NaCl background using three identical ITO-POT-membrane electrodes. Scan rate: 100 mV s^{-1}.

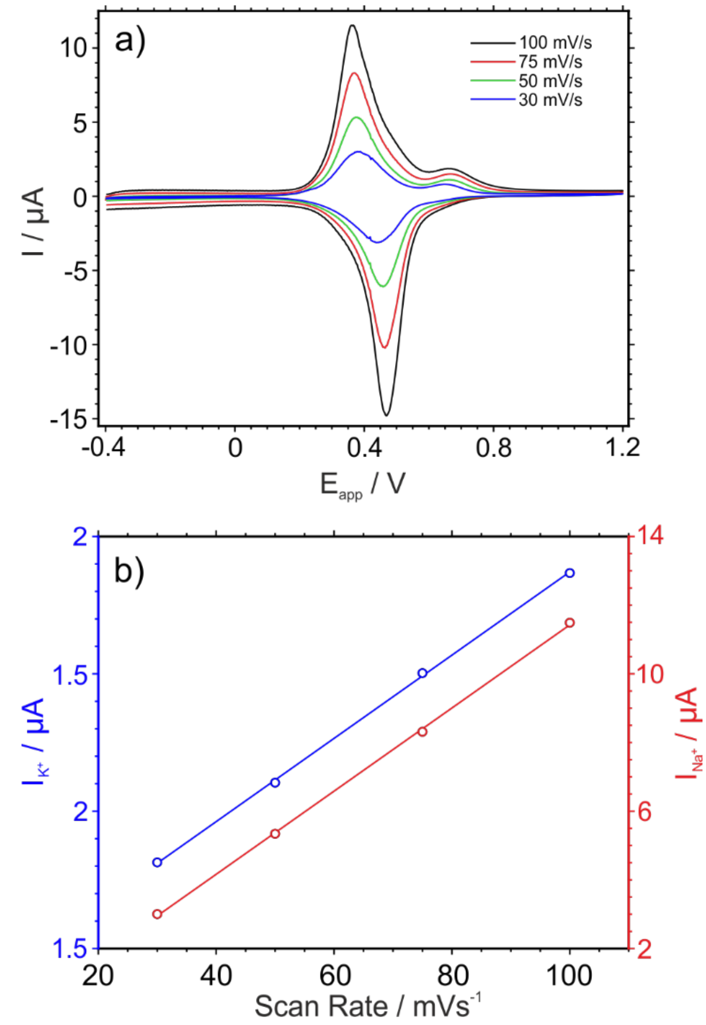

Figure S5. (a) Cyclic voltammograms observed for 5 μM KCl concentration in 10 mM NaCl solution at increasing scan rates of 30, 50, 75, 100 mV s^{-1}. (b) Linear relationships between the peak current of Na^{+} and K^{+} with the increasing scan rates.

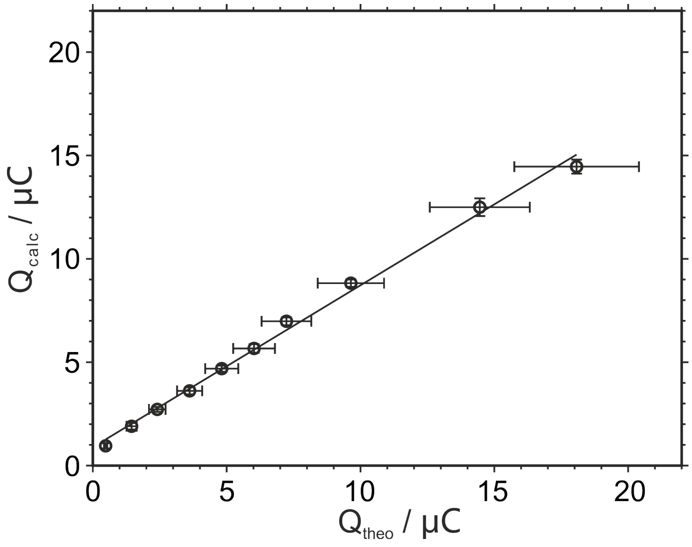

Figure S6. Correlation between the calculated charges (n=3, Q_{calc}) and the theoretical charges (Q_{theo}) present in thin layer samples containing increasing K^+ concentration from 1 to 37.5 μM. The vertical error bars indicate the standard deviation observed between the three electrodes. The horizontal error bars represent the uncertainties associated with the theoretical charges' calculation.
